# Interprofessional versus uniprofessional dyad learning for medical students in a clinical setting

**DOI:** 10.5116/ijme.5f50.bc76

**Published:** 2020-09-28

**Authors:** Torben Bæk Hansen, Britta Pape, Pernille Staal Thiesen, Flemming Jakobsen

**Affiliations:** 1 University Clinic for Hand, Hip and Knee Surgery, Regional Hospital West Jutland, Denmark

**Keywords:** Dyad, uniprofessional, interprofessional, clinical learning, indirect supervision

## Abstract

**Objectives:**

The aim of the present study was to explore and compare medical students' perceived learning outcomes when treating patients under supervision in two different learning settings: a uniprofessional or an interprofessional dyad.

**Methods:**

The design of the study is a qualitative interview study. Data were collected from October 2016 to June 2017 via semi-structured group interviews performed at the end of the clinical placement in an orthopaedic outpatient clinic for medical students in the last semester of the curriculum. In the placement, the students worked by turns in either a uniprofessional dyad with two medical students or an interprofessional dyad with a nursing student. The data from the interviews were analysed using Systematic Text Analysis.

**Results:**

Overall, 21 students were interviewed. The students appreciated the authenticity of dealing with real patient problems. Both dyads provided the possibility of working as a professional, but the interprofessional dyad had a more authentic setting. In both dyads, the students' interdependence and mutual support promoted the acquisition of knowledge and skills. Working in the interprofessional dyad facilitated relationships between the professions, and the medical students became aware of some of their own profession's strengths and weaknesses. The interprofessional collaboration contributed to different perspectives on the patients' course of treatment and led to a more holistic understanding of the treatment.

**Conclusions:**

Interprofessional dyads have the potential to improve learning outcomes in the clinical training of medical students. Further studies are needed to explore the benefits across medical specialities and settings.

## Introduction

Learning in a clinical environment is motivating for learners because of the authenticity of working with real patients with real problems in hospital wards.^1^ However, fewer hospitalised patients and more ambulatory patients^2^ means that medical students' clinical education, to a greater extent, must be moved from the hospital wards to the busy outpatient clinics ^3^, with unpredictable and challenging situations that can give rise to tension between the clinical supervisor's dual role as both a clinician and teacher.^4^

This is challenged by the students' perspective, where they want more patient-based learning and teaching, including self-directed learning and formative assessment of clinical skills.^5^ One way to improve the learning environment is to use peer learning, where students work in pairs (dyads) with a student colleague from the same profession (uniprofessional)^6^^,^^7^ or with a student from another profession(interprofessional).^8^ The goal of peer learning is the acquisition of knowledge and skills by helping and supporting each other. One type of peer learning is cooperative learning, which can be described as 'structuring positive interdependence',^9^ where the students have a common goal, such as the removal of sutures and checking the range of motion in an orthopaedic outpatient clinic. This method, where students with the same level of education work and learn together, has been described in different settings.^10^^,^^11^ Tai and colleagues found that medical students in their first clinical year used peer-assisted learning in formal and informal situations and found it useful.^6^ Dyad training, where medical students work with simulated patients and train patient encounter skills, was effective, efficient and demonstrated higher confidence in managing patient encounters compared to training alone.^12^ However, questions have arisen concerning

dyad training involving real patients, and research concerning the use of dyadic training for more advanced clinical students in the clinical environment is needed.^13^

For many years, clinical learning for medical students has taken place in interprofessional training units, where students from different professions work and learn together in a clinical setting. The most widely used professions have been nursing, physiotherapy and occupational therapy students. ^14^^-^^20^ Results have indicated that the interprofessional learning environment contributes to the students' formation of professional identity, uniprofessional learning, interprofessional learning, and learning about interprofessional collaboration. ^21^^,^^22^ Similar positive results have been reported from interprofessional clinical learning in outpatient clinics.^23^^-^^25^

To our knowledge, a comparison of uniprofessional dyadic training versus interprofessional dyadic training of medical students in an authentic setup in a busy outpatient clinic has not been described. Based on the current literature, dyadic training seems to have the potential for peer learning in both situations. Still, we hypothesise that the learning outcomes in the two settings may be different. Thus, this study aimed to assess medical students' perceived learning outcomes in a uniprofessional versus an interprofessional dyad treating patients in an orthopaedic outpatient clinic.

## Methods

### Setting

The study was conducted in an orthopaedic outpatient clinic, where patients came for follow up after surgery or after initial treatment in an accident and emergency clinic. In the morning on two weekdays during a five-week clinical placement, one 12th (of 12) semester medical student and one 6th (of 7) semester nursing student examined and cared for three patients per day (interprofessional dyad). As we wanted to compare this setting with uniprofessional dyad training, two students from the same group of medical students on two other weekdays examined and cared for three patients per day in a uniprofessional dyad. Therefore, during their clinical placement, all medical students participated in both scenarios. In both scenarios, we chose uncomplicated patients (e.g., removal of cast and sutures followed by an assessment of the range of movement) with medical problems that were within the expected clinical knowledge and skills of the students. The patients were identified on the day before the consultation by the clinical tutors (surgeon or nurse) so that the students could prepare themselves for the consultations. In the morning, before the students performed their consultations, they accounted for their plans and had the chance to have necessary supplementary indirect supervision from their supervisor(s). A surgeon and a nurse supervised the interprofessional dyad, and a surgeon supervised the uniprofessional dyad. The rest of the morning was similar for the two dyads. After the indirect supervision, the students went to their assigned room, where they made final preparations for their consultations, including the distribution of roles. In the uniprofessional dyad, they alternated between being responsible for the consultation and being the observer, while in the interprofessional dyad, they distributed the tasks according to their professional knowledge and capability. The students were alone with the patients but were able to call for a supervisor at all times. In the uniprofessional dyad, the medical students had the possibility of support from a trained nurse or a surgeon, but the consultation was performed without a nurse, and the nurse did the nursing tasks (e.g., casting and wound dressing) afterwards. After performing their consultations, the students received individual indirect supervision with feedback on the consultation from a surgeon or a nurse, depending on their profession.

As a pedagogical approach, we used Knowles's principles for Adult Learning as guidelines.^26^^-^^30^ Furthermore, we supplemented the interprofessional dyad with the Contact Hypothesis as a guiding principle for the supervision.^31^^,^^32^

### Design

As we wanted to explore medical students' experiences from their clinical placement in the orthopaedic outpatient clinic, we chose an explorative qualitative single-case study with two embedded units of analysis: the students' experiences in the uniprofessional dyad and their experiences in the interprofessional dyad.^33^

### Data collection

In the period from October 2016 until June 2017, we had six five-week placements for medical students in their final year. During the period, 21 students participated in both the uniprofessional and interprofessional dyads, and this was considered a convenient sample size for a qualitative focus group study. All students were included in the study, resulting in six semi-structured focus group interviews with two to six students in each group. The interviews were performed at the end of the clinical placement and took place in the orthopaedic surgeons' conference room. The mean duration was 50 minutes (range 38 – 60 minutes). Authors BP and PST alternately performed three interviews, with one as the interviewer and the other taking notes and asking investigative questions.

The interview guide had nine questions about both dyads (Table 1). During the interviews, all group members were encouraged to elaborate on their answers. A secretary transcribed the interviews, and the transcription was immediately reviewed by the interviewer to check for misunderstandings.

#### Data analysis 

For analysis, we used the qualitative, descriptive and explorative method of Systematic Text Condensation.^34^ Authors BP and PST began the analysis by individually reading through the transcripts to obtain a general impression of the material and to look for preliminary themes associated with the medical students' perceived learning outcomes in the interprofessional and uniprofessional dyads. This was followed by a discussion of confluent and divergent themes. The next step was to read through the text again in search of meaning bearing units. The meaning bearing units were decontextualised, marked with codes, and copied into a new document. The coding took place as a flexible and iterative process because some of the preliminary codes turned out to not sufficiently describe what we were looking for. Next, the decontextualised meaning units were sorted into final themes – again as an iterative process, where necessary re-coding could take place. The last step of the analysis was synthesising the transformed meaning units into consistent statements regarding the medical students' experiences. 

#### Ethical considerations

According to the Danish National Committee on Health Research Ethics, studies based on questionnaires or interviews are exempt from approval by the committee.^35^ All students volunteered, were informed of the project, and accepted that their statements would be presented anonymously. All person-related and demographic data on the students in the interviews were destroyed after the transcription of the focus group interviews.

**Table 1:** Focus group interview guide

1	What was your learning outcome?
2	What do you think you have learned from working together with another medical student or a nursing student?
3	Do you think that the medical/nursing student had expectations of you – and could you fulfil those expectations?
4	What do you think of responsibility and role distribution in the two dyads?
5	What was the relevance of collaborating with another medical student or a nursing student?
6	Did anything in the collaboration surprise you?
7	Do you think differently about your own profession after this learning experience?
8	What has the pedagogical approach meant for your learning outcome?
9	Can you use what you have learned in the two settings in your future work as a doctor?

## Results

The analysis identified four major themes that resonated across all six interviews with subthemes (Table 2).

### Discovering conditions for learning

#### Authenticity

One of the major issues the medical students pointed out in the study was the experience of authenticity when dealing with real patient problems in a safe learning environment. A student in the focus group interview 4 said:

"This is one of the clinical placements I have made the most of. You get the chance to treat your own patients in a safe learning environment. Now I am more ready to be a doctor than I was, when I arrived four weeks ago".

There appeared to be a difference between the two dyads' learning environments, where the uniprofessional dyad was described by some students as a fictional constellation, and a student in focus group interview 6 said:

"I think the uniprofessional dyad is fictional and that constellation will never appear in real life. But collaborating with a nurse, that will happen very, very often".

Nonetheless, the medical students expressed that they could train their ability to think autonomously and to work unassisted on patient-based tasks in both dyads.  A student in focus group interview 4 said:

"In our former clinical experiences, we have been like a dog on leash looking passively on while the doctor handled the consultation".

Thus, regarding authenticity, both dyads provided the medical students with the possibility of working as a professional, but the interprofessional dyad had a more authentic setting.

#### Indirect supervision

Indirect supervision prior to seeing patients made the medical students feel well prepared for the autonomous handling of patient-based tasks during the consultation and contributed to increasing their learning outcomes. A student from focus interview 5 said:

"They try to prime you because they count on you to manage the task. The trust that you can grow with the task".

The indirect supervision also made it clear when to call for supervision during the consultation and prepared the students to handle a considerably larger amount of doubt. The medical students felt that reflecting on and discussing the patients' course of treatment in the indirect supervision before and after the consultation was an educational experience, supplemented with feedback on their notes in the patients' records. A student from focus group interview 1 said:

"Because of the supervision on the notes, you get better off; it is hugely important that we do not get used to bad habits because, until now, no one told us what was wrong and what was right".

Contrary to the uniprofessional dyad, the medical students felt that, in the interprofessional dyad, all aspects of the patients' course were considered and discussed in the indirect supervision, and a student from focus group interview 3 said:

"Participating in the interprofessional supervision gives a better overall picture. You get a better general impression of the patient's situation".

Therefore, regarding indirect supervision, we found that indirect supervision contributed to supporting and improving students' learning outcomes in both dyads. The students found it educational to explicitly discuss professional knowledge and skills with the supervisors. In addition, they found a difference in the understanding of the patient in the two dyads. During the indirect supervision in the uniprofessional dyad, they realised that their focus was on professional knowledge and skills, whereas in the interprofessional dyad, the indirect supervision also provided a more general impression of the patient's situation.

**Table 2:** Themes and subthemes

Discovering conditions for learning	Appreciating the value of uniprofessionalism	Appreciating the value of interprofessionalism	Discovering professional identity
Authenticity	Cooperative learning	Cooperative learning	Working independently
Indirect supervision	Professional knowledge and skills	Learning about the other profession	Give-and-take responsibility
Learning about the organisation		Holistic understanding for the patient's course of treatment	Reflection on each other's role

#### Learning about the organisation

Because the patients and the organisation in both dyads depended on the students' presence and efforts, the medical students felt they provided a useful function. They pointed out the importance of taking an active part in the consultations because they explained that, when they were relieved of their passive role and allowed to take control, they felt a commitment and reflected on how and with whom the problem could be solved. The medical students felt they increased their understanding of the need for collaboration with other professions and with an efficient workstream to practice the best course of treatment for the patient in the organisation. A student from focus group interview 4 said:

"When you have to be more active, and when collaborating with other professions, you are forced to think about who I should contact to solve this problem and how does it work organizationally".

The medical students found it reassuring and important that the organisation provided scheduled opportunities for learning. As there is a risk of indirect supervision and an educational approach being random and relying on an individual surgeon, the medical students experienced that maintaining a positive learning environment could be vulnerable, as stated below by a student from focus group interview 2:

"If just one of the supervisors is absent, then nobody else can step in, and it will just be cancelled".

Thus, regarding the aspect of learning about the organisation, the students gained insight into the operation and maintenance of the organisation and experienced first-hand the complexities of patient-based problem-solving. The learning outcome was influenced by the organisation's prioritisation and maintenance of the learning environment.

### Appreciating the value of unprofessionalism

#### Cooperative learning

In the uniprofessional dyad, the students valued the feeling of equality, collaboration and professional discussions, including the sharing of knowledge and experiences with their student colleague. At the same time, according to the students, this gave them a certain confidence in the process of becoming a doctor. They built up medical knowledge and they felt relieved when they realised that the other medical student could also be in doubt about the treatment of patients. A student from focus group interview 3 said:

"It has given something extra having a partner for discussion at the same level as myself – we have learned from each other's experiences".

A student from focus group interview 4 said:

"It is nice to see that others can make a mess of things and to know that it is not me that is lost behind a cart".

In the interprofessional dyad, the medical students also felt equality in their collaboration. Both students were there to learn. They felt they could have an equal dialogue with the nursing student, as illustrated in the following statement by a student in focus group interview 3:

"Well, you know something, I know something else, and none of us says this is the answer book. Together we are working towards what we think is the correct solution".

Therefore, regarding uniprofessional cooperative learning, we found that the students' interdependence and mutual support promoted the acquisition of knowledge and skills in both dyads. However, there seemed to be a difference in their perception of equality in the two dyads. In the uniprofessional dyad, equality was concerning professional knowledge and skills, and in the interprofessional dyad, equality was concerning students.

### Professional knowledge and skills

In the uniprofessional dyad, the medical students reflected on the professional knowledge and skills and not on their

communication with the patient. A student from focus group interview 2 said:

"We talked about the decisions I had made – not so much about communication – but more about the professional part – what he thought about it".

They felt that the professional discussion helped to practice their ability to argue in favour of their professional actions and decisions. A student from focus group interview 1 said:

"You learn from asking each other questions and then try to read about it. You consider things a little bit more when being forced to find out why things are as they are".

In the interprofessional dyad, the medical students felt that they could not discuss specific medical issues with the nursing student. A student from focus group interview 1 said:

"The downside is, that you do not have a colleague for discussion about the specific medical issue".

Thus, regarding professional knowledge and skills, we found that there was a distinct difference between the two dyads. In the uniprofessional dyad, they discussed with each other and felt a considerable larger development in their specific medical knowledge and skills than in the interprofessional dyad.

### Appreciating the value of interprofessionalism

#### Cooperative Learning

In general, the medical students described a lack of attention to interprofessional collaboration both at the university and in their former clinical training. When working in the interprofessional dyad, they enjoyed the collaboration and became aware of the importance of facilitating interprofessional relationships in their future work as doctors. A student from focus group interview 3 said:

"At the medical faculty, we are taught to be loners".

A student from focus group interview 1 said:

“All others, nurses, physiotherapists and occupational therapists are together. You have to get used to a good contact because they are the ones you are going to collaborate with".

The interprofessional dyad provided an opportunity for developing professional friendships, and some medical students discovered that nursing students were nice and pleasant to collaborate with. A student from focus group interview 4 said:

"It was surprisingly cozy. They were not that harsh; it was really good".

Furthermore, they found that with the help of mutual discussion, where both the nursing student and the medical student were dependent on the other's professional knowledge, they were able to decide independently of the supervisors. They explained that they realised that the responsibility for the patient's course of treatment was shared between the individual professional and the team. A student from focus group interview 3 said:

"The purpose is to be aware of the different professions' substance and that they all only are worthwhile if we collaborate".

Thus, regarding interprofessional cooperative learning, we found that working in the interprofessional dyad facilitated relationships between the professions. Sharing different kinds of professional knowledge and responsibilities was essential for cooperative learning and for making decisions relating to the patients.

### Learning about the other profession

After working in the interprofessional dyad, the medical students became aware of the importance of both being precise in their communication and acting professionally toward each other while treating patients. In that way, there was no doubt as to who performed the different tasks. A student from focus group interview 6 said:

"When talking with the nurse in the consultation with the patient present, it is important to use a good tone and to be professional to each other. It is important to have a common starting point and to agree on the distribution of roles".

In the interprofessional dyad, the students represented different professional skills. The medical students were surprised that the nursing students had more and wider professional skills than they expected. It became more obvious for the medical students how they, together with the nursing students, could optimise each other's professional skills and knowledge in their future work. A student from focus group 4 said:

"The nurses had more and wider competencies than anticipated. That is something you have to learn. It is a positive surprise to learn what they can and what we can".

Therefore, regarding learning about the other profession, we found that, when working in the interprofessional dyad, the medical students learned about mutual respect and about being accurate in their communication with other professions. They learned how the two professions' skills complemented each other and contributed to the treatment of the patient.

### Holistic understanding of the patients' course of treatment

The medical students explained that they were normally focused on their professional role and on being accurate in their professional knowledge and skills, for example, in interpreting an X-ray. They found that the nursing students were simple and practice-oriented in their approach to the patients. The responsibility to communicate with the patients could benefit from being shared with the nursing student in tasks involving patients coping with their daily life. A student from focus group interview 2 said:

"The nursing student had a wider focus than I had. This opened my eyes for the importance of not only examining the x-ray and range of motion but also taking into account the importance of the patients' activities of daily life".

Regarding holistic understanding of the patients' course of treatment, we found that, by participating in the interprofessional dyad, the medical students became aware of some of their own profession's strengths and weaknesses. Interprofessional collaboration contributed different perspectives on the patients' course of treatment and developed a more holistic understanding of that treatment.

### Discovering Professional identity

#### Working independently

The medical students reported that, when they were on their own in the uniprofessional dyad, they felt more as a doctor than a medical student, as expressed in this statement from a student in focus group interview 6:

"When there is a supervisor present – where we should do the talking – we do not appear like real doctors – because he is standing in the background – and the patient knows it". 

If the nursing supervisor was present in the interprofessional consultation, attention and authority could be drawn away from the medical student, whereby that student missed opportunities to expand their patient-based learning. A student from focus group interview 3 said:

"It matters if the nursing supervisor is present all the time because it moves focus from medicine to nursing".

Thus, regarding working independently, the presence of supervisors in the consultation room minimised the medical student's possibility of developing a professional identity.

### Give-and-take responsibility

A few medical students reported that they experienced more responsibility in the uniprofessional dyad than in the interprofessional dyad. Their experience was that the assigned types of patients in the uniprofessional dyad were more "complicated" medically. This is contrary to the interprofessional dyad, where they sometimes felt their presence was unnecessary because the nursing student had the skills to handle the situation. Thus, they experienced less professional responsibility when collaborating interprofessionally and were mere onlookers. A student from focus group interview 4 said:

"You feel that the nursing student has most of the responsibility and is entirely in control of the situation; she can remove the sutures, and she takes care of what I am not in control of".

However, several other students reported they felt more as a doctor in the interprofessional dyad because they had to make decisions on their own, just like a doctor, contrary to working in the uniprofessional dyad, where there were two medical students working together and sharing the responsibility. They explained that, even though they were able to discuss the situation with the nursing student, it was their responsibility as doctors to make a decision for treatment. A student from focus group interview 4 said:

"If the nursing student has asked me, what do you think of this issue, I feel I have a responsibility for being able to answer. If I could not answer, it was my responsibility to call a surgeon".

Therefore, regarding give-and-take responsibility, the feeling of trust and responsibility was essential and provided a state of mind that included confidence, motivation and a sense of professional identity. In the interprofessional dyad, the feeling of responsibility was dependent on the assigned types of patients.

### Reflection on each other's roles

Several students reported that, in the uniprofessional dyad, they learned from observing the other medical student's ways of performing professional skills. By means of common reflection after they had seen the patient, they got new ideas for performing their professional role but also witnessed ways they did not wish to replicate. A student from focus group 1 said:

"It is always exciting to observe how my student colleague is performing. Things can be conducted in different ways, and we can give each other competent feedback on our professional role".

A student from focus group interview 5 said:

"Sometimes you might think that I would not have done it in that way".

In the interprofessional dyad, they reflected on their professional role by observing the differences among professions. When working together with a nursing student and observing their performance and skills, they became aware of knowledge and skills different from their own, as expressed in the following quotation by a student in focus group interview 1:

"In the interprofessional dyad, it is very clear who does what. I have got a better understanding both of their professional role and my own professional role".

Thus, regarding reflection of each other's roles, we found that, when working in the uniprofessional dyad, the medical students learned about professional roles by observing and relating to the other medical student's performance. In the interprofessional dyad, they observed and related to the differences among the professions and thereby became more aware of the professional roles. Both dyads promoted their sense of professional identity.

## Discussion

The most important finding in this study was that the medical students achieved valuable learning in both the uniprofessional and interprofessional dyads, but the learning outcomes were different.

The students described the learning environment as an essential prerequisite for their learning outcome. We used the seven principles described in Knowles' principles for Adult Learning.^26^^-^^30^ In our learning environment, the supervision was as indirect as possible in both dyads before and after the students observed the patients. The students were expected to be at the forefront and well prepared so that they could present a plan for the consultation at the supervision before they observed the patient. This approach lived up to 'Learners need to know the relevance of what they need to know' because, when the students read the patient's record the day before the consultation, they became aware of what they needed to know to handle the consultation. Being alone with the patients and being considered responsible and capable of making their own decisions was in accordance with 'Preferring responsibility for their decisions'. As for 'Accumulation of experience', it is also important to remember that the students were at different clinical, theoretical and personal levels^36^; therefore, the individualisation of supervision and learning strategies was necessary. 'Readiness to learn' was fulfilled because the necessity of learning what they need to know is a consequence of the pedagogical approach, where the students will undertake consultations unassisted. 'Orientation to learning' falls directly and precisely into this setting because the students were expected to have a task-centered orientation with a direct focus on the patient's problem. 'Students can work collaboratively and in dialogue with others with mutual trust and respect' was fulfilled because, according to the students, they worked collaboratively and in dialogue with each other and with their supervisors in both of the dyads. Lastly, 'Adult learners are responsive to some external motivators, but their most potent motivators are internal'; in this setting, the students' internal motivators showed that they were accountable for completing the consultation together with their colleague.

Authenticity in both the uniprofessional and interprofessional dyads when communicating directly with real patients with real problems in a safe learning environment made the students feel more prepared for their future role as doctors. However, even though the students said they had acquired professional knowledge and skills in the uniprofessional dyad, some of them found this dyad to be unauthentic because that constellation would never occur in real life. Dyad training for medical students has typically been assessed in simulation settings, and it has been questioned how this form of training can be transferred to real-world settings.^37^ Statements from some of the students in this project are a reminder to avoid unauthentic scenarios as much as possible.

Even though the preferred supervision was indirect, there were also situations where direct supervision was needed. However, the students pointed out that it was important to avoid having supervisors in the room when they were together with a patient because the patient's focus was moved from the student to the supervisor, and the students experienced a decrease of autonomy.^38^

According to the students, the organisation gave priority to the learning environment, but they also expressed awareness that this priority was resource-demanding and fragile because of the dependence on the affiliated supervisors' attendance, as described by Dent.^3^

As a subtheme under 'Uniprofessionalism', we find 'Cooperative Learning', where the students had the shared goal of learning from each other by shifting between observing and performing patient consultation and afterward discussing the course of the consultation.^9^ In this study, the medical students reported no concerns about missing opportunities for hands-on training skills, as reported in one study about clinical dyad training for nursing students.^11^ The other subtheme, 'Professional knowledge and skills', included the development of professional competence and communication with the patient. The students' feeling of equality in their uniprofessional discussions before and after they observed the patient contributed to developing their professional knowledge and skills. They felt that they learned from asking each other questions and from discussing medical issues relevant to the patients' condition. This finding is in line with a study on case scenarios, which revealed that dyad training in managing patient encounters was superior to training alone.^12^ From the findings, it is clear that, in the uniprofessional dyad, the students concentrated on the medical aspects, contrary to the interprofessional dyad, where the medical students missed the opportunity to discuss specific medical issues. Despite this missed opportunity to discuss strictly medical topics, the medical and nursing students complemented each other during the consultation and worked toward what they thought was the best solution for the patient.

### Interprofessional

The interprofessional dyad lived up to the assumptions of the Contact Hypothesis.^31^^,^^32^^,^^39^ There was equality between the students in this situation because both could contribute when they presented their plans to the supervisors and when they jointly performed their consultation. Their common goal was to help the patient. They collaborated in the consultation and offered perspectives from the two professions, being aware that they could call for a supervisor if necessary. Lastly, there was a friendship potential, as illustrated by the medical students saying that the nursing students were comfortable to work with. This finding is in line with our findings in an earlier study from the same setting^40^ but in opposition to other researchers who found difficulties in collaboration with hierarchical verbal dominance and challenges concerning status in teams among post-graduate health professionals.^41^^-^^44^ This opposition between pre- and post-graduate findings underpins the importance of interprofessional undergraduate education to obtain students' feeling of equality, including shared team identity, clear roles, integration, interdependence and shared responsibility.^45^

The goal in the uniprofessional dyad under the subtheme' cooperative learning' was common learning. In the interprofessional dyad, the common goal was to help the patient and thereby learn about each other's profession. The students were interconnected and felt a positive interdependence working face-to-face, with individual accountability for the patient, and they followed up after the consultation with a common reflection on how they both performed and thereby trained their social skills.^46^

Therefore, the outcome resulting from cooperative learning in the interprofessional dyad was different from that in the uniprofessional dyad. The medical students said that there had been a lack of attention to interprofessional issues in their former clinical training, where they were trained to be loners, but in the interprofessional dyad, they became aware of the value of different perspectives on the same issue. Indeed, when they observed the patient together, they did not see the same things.^47^ They learned that cooperative learning, involving sharing different perspectives and professional knowledge, made it easier to make decisions for the patient. ^48^ The medical students' existing stereotypes about nursing students were challenged because they found that the nursing students had wider competencies than expected; this experience altered their attitude toward nursing students in general, as described in the literature.^24^^,^^25^^,^^39^ Furthermore, by observing the nursing students' focus on the patients' activities in daily life, the medical students broadened their picture of the patient, from examining only X-rays and range of motion to having a more holistic view about what is important for patients in their daily life, e.g., how much weight the patient was allowed to carry when going shopping.

Professional identity. Working independently without a supervisor present resulted in the students feeling more like doctors and less like medical students. According to Kogan et al.^49^ direct observation of clinical encounters is a key strategy. However, as mentioned above, in this learning environment, the presence of supervisors in the consultation room, where the patients' focus was moved from student to supervisor, could minimise the medical students' possibility of developing a professional identity.^49^

The medical students presented different opinions on the impact of interprofessional dyad on their formation of professional identity. A few of the students reported that they felt that their presence in the interprofessional dyad was unnecessary because the nursing student had the skills to handle the situation, making the students feel like onlookers. This situation could indicate that the supervisors had not been aware of selecting patients, which provided a challenge for both the medical and nursing students, as recommended by D'Eon.^46^ As opposed to this experience, several other students reported that only the interprofessional dyad made them feel more as doctors because they alone were accountable for the medical decisions.

In both dyads, the medical students reflected on their student colleague's role, but there was a difference in their focus in the two dyads. In the uniprofessional dyad, they focused primarily on the other medical students' performance of medical actions and learned that things could be done differently. This is in line with the findings of Tolsgaard and colleagues^13^ who reported that (uniprofessional) dyadic clinical skills training provided useful insights through observation.

Earlier studies about interprofessional education have described that understanding and appreciating others' roles, together with effective formal and informal communication, are important ingredients in interprofessional education.^50^^, ^^51^ These findings are in line with the findings in this study, where the medical students expressed that they became aware of the differences in the two professions in the interprofessional dyad and thereby increased their awareness of their own professional role as future doctors.

A limitation of this study is that all findings were self-reported, which may imply a risk of social desirability bias. To avoid this bias, researchers without direct affiliation to the outpatient clinic performed the interviews. Another limitation is that all findings in this study derived from the same setting of only a few participants; this fact may reduce transferability to other settings. We could have chosen a structured observational evaluation of the consultation, but this would have influenced the authenticity of the setting.

In spite of these possible limitations, we believe that this article adds to the published literature about clinical training in dyads and, as such, can be useful as an inspiration for establishing more uniprofessional and interprofessional clinical placements in ambulatory settings.

## Conclusions

There were some similar learning outcomes in the uniprofessional and interprofessional dyads. In both dyads, medical students improved their professional knowledge and skills. They obtained insights into the operation and maintenance of the organisation and learned about the complexity of solving patients' problems. The students appreciated the authentic learning environment with indirect supervision. Furthermore, the students also noted the importance of the organisation's prioritisation of the learning environment.

Interestingly, there were also different learning outcomes in the two dyads. In the uniprofessional dyad, the learning outcome was primarily medical, while in the interprofessional dyad, medical students learned about a more holistic approach to the patient's course along with learning to collaborate interprofessionally.

Therefore, we find that interprofessional dyads seem to have the potential for improving learning outcomes in the clinical training of medical students. However, further studies are needed to explore the benefits across medical specialities and settings.

### Acknowledgements

The authors would like to thank the students for their willingness to be interviewed.

### Conflict of Interest

The authors declare that they have no conflict of interest.
